# Social anxiety disorder and its associated factors: a cross-sectional study among medical students, Saudi Arabia

**DOI:** 10.1186/s12888-022-04147-z

**Published:** 2022-07-27

**Authors:** Wejdan M. Al‑Johani, Nouf A. AlShamlan, Naheel A. AlAmer, Rammas A. Shawkhan, Ali H. Almayyad, Layla M. Alghamdi, Hatem A. Alqahtani, Malak A. Al-Shammari, Danya Mohammed Khalid Gari, Reem S. AlOmar

**Affiliations:** 1 Department of Family and Community Medicine, College of Medicine, Imam Abdulrahman Bin Faisal University, P.O. Box 1982, Dammam 34224, Saudi Arabia; 2 College of Medicine, King Khalid University, Abha, Saudi Arabia; 3 College of Medicine, Imam Abdulrahman Bin Faisal University, Dammam, Saudi Arabia

**Keywords:** Social phobia, Social anxiety disorder, Prevalence, Medical students, Kingdom of Saudi Arabia

## Abstract

**Background:**

Social Anxiety disorder (SAD) is common worldwide. However, data from Saudi Arabia is deficient. This study aims to determine the prevalence of SAD across Saudi medical students and its associations with sociodemographic factors and their academic performance.

**Methods:**

The main outcome was presence/absence of SAD and the secondary outcome was its level of severity. These were assessed from the Social Phobia Inventory. Associated factors included sociodemographic variables, as well as educational characteristics of students. Descriptive statistics were reported as counts and percentages, and unadjusted and adjusted odds ratios (OR) and their 95% confidence intervals (CIs) were computed through bivariate and multivariate logistic regression.

**Results:**

Of 5896 Saudi medical students who participated in the study**, **the prevalence of SAD was almost 51%. While 8.21% and 4.21% had reported severe and very severe SAD, respectively. Older age students were at lower risk of developing SAD (OR = 0.92, 95% CI = 0.89 – 0.96). In contrast, females (OR = 1.13, 95% CI = 1.01 – 1.26), students enrolled in private colleges and colleges implementing non-problem-based learning (OR = 1.29, 95% CI = 1.09 – 1.52 and OR = 1.29. 95% CI = 1.15 – 1.46 respectively) were at higher risk. A significant elevated risk of SAD was found among students who had previously failed, and had a low GPA.

**Conclusion:**

SAD is prevalent among the sampled population, and different associated factors were identified. Current results could raise the awareness of faculty members and healthcare providers towards early detection and management of these cases.

## Background

Social Anxiety Disorder (SAD) which was initially named social phobia, is defined according to the Diagnostic and Statistical Manual of Mental Disorders (DSM-5) as an extreme fear or anxiety about one or more social situations in which the individual is exposed to scrutiny by others, for instance, social interactions (e.g., meeting and talking to new people), being observed (e.g., eating or drinking), and performing in front of others (e.g., public speaking) [1]. The most common reported presentation of SAD was fear of speech-making [2]. SAD can occur in any public place where a person feels observed and judged by others [3]. Individuals will develop different cognitive and somatic anxiety symptoms characterized by autonomic stimulation, such as blushing, tremors, increased sweating, and tachycardia [4].

It is one of the most predominant anxiety disorders among adolescents and younger aged groups, impairing their functioning capabilities if left untreated [5]. In addition, its high prevalence would make it the third most common mental disorder after depression and alcohol abuse [6].

The lifetime prevalence of SAD has been reported in various studies, ranging between 3–13% [1]. The prevalence of SAD among university students has been assessed in multiple studies. In Jordan, Ghana, Nigeria, Brazil and Sweden Universities, the prevalence was around 9–16.1% [5, 7–10]. A higher prevalence of SAD has been found among university students in Ethiopia and India (26%, 31.1%) respectively [11, 12]. Moreover, it was associated with female gender, low educational attainment, positive personal or family history of mental disorders, psychiatric medication use, and lack of social support [2, 13]. Studies have shown that SAD has led to low self-esteem and impaired body image, consequently negatively impacting on students' academic performance [14, 15].

Furthermore, SAD is considered a significant risk factor for developing major depressive disorders and alcohol abuse disorder [16].

Although various studies worldwide have assessed the prevalence and impact of SAD among different populations, Saudi Arabia's data is scarce. After a thorough literature search, few data regarding SAD among medical students in small cities in Saudi Arabia have been obtained. Medical students are more exposed to academic challenges, including the lengthiest education and training period, the stress of multiple written and clinical examinations, oral presentations, interaction with patients and their families, and exposure to serious life and death issues. Consequently, medical students particularly require intact physical and mental well-being, strong personality structures, and a willingness to attain professional and communication skills to deal with academic challenges [17]. Therefore, this study aims to estimate the prevalence of SAD among medical students in the Kingdom of Saudi Arabia (KSA) and determine its association with students' sociodemographic factors and academic performance.

## Methods

### Study design and participants

This cross-sectional study included all medical students and medical interns both males and females attending any medical college in Saudi Arabia whether private or governmental. The number of medical colleges in Saudi Arabia is rising to 34 colleges, 27 of which are governmental. All Saudi medical colleges provide six-year undergraduate study, followed by one year of practical internship [18, 19].

### Sample size and sampling technique

The Saudi Commission for Health Specialties in its most recent published report stated that the total number of undergraduate students in medical colleges both private and governmental was 101,256 students [20]. The minimum required sample was calculated to be 2342 students using Epiinfo V.7.0. The 51.9% of presence of (SAD) was obtained from a Saudi study that examined social phobia among Saudi students in a single college, with an alpha level of 0.05 and a precision of 2% [21].

A non-probability sampling technique was used where students were invited to take part by answering an online-based questionnaire. The QuestionPro questionnaire software (Seattle, Washington, USA) was used.

### Data collection tool and processes

Data were collected using a validated online self-administered questionnaire consisting of two parts. The first part included the socio-demographic information (age, gender, educational level, marital status, income, and Grade Point Average (GPA)). The second part included the validated Social Phobia Inventory (SPIN) questionnaire by K. M. Connor, a screening tool for SAD, consisting of 17 items. Each point is ranked with a five-degree Likert scale (0 = No, 1 = Low, 2 = Somewhat, 3 = High, 4 = Very Much). The total score ranges from 0 – 68; thus, an individual who scores more than 20 is considered to have SAD. The SPIN had good test–retest reliability, internal consistency, convergent and divergent validity, the Cronbach alpha is 0.85. Therefore, SPIN can be used as a measurement for the screening of SAD and monitoring the responses of treatment [22, 23]

The online link of the survey was sent to the students' phone numbers through assigned data collectors from each college. The survey was customized to accept a single response from each number to avoid duplication of responses.

### Statistical analysis

The primary outcome in this study was whether medical students had SAD or not according to the Social Phobia Inventory. A secondary outcome is the severity of SAD which may be computed from the inventory itself. After summing all 17 items of the inventory, participants who score less than or equal to 20 are assumed to not have SAD while those who score above 20 do have SAD. As for the severity as a secondary outcome, a participant scoring from 21 to 30 is considered to have mild SAD, 31 to 40 as moderate, 41 to 50 as severe and more than 51 as very severe. Descriptive statistics were obtained by counts and percentages, and potential associations were tested through the Pearson’s *X*^2^ test and the T-test. Trends of proportions over GPA were tested for statistical significance. Unadjusted and adjusted Odds Ratios (ORs) and 95% Confidence Intervals (CIs) were drawn through binary logistic regression analyses where the outcome was for the presence/absence of SAD. Final variables in the regression model were decided based on a Directed Acyclic Graph of associations and were not entirely based on significance testing of bivariate associations. The model with the best fit was chosen based on model diagnostics. The Variance Inflation Factor measure was used to test for multicollinearity. All analyses were performed in Stata V.15.0.

## Results

### Characteristics of the students

A total of 5896 students participated in this study (5.82% of the target population). It included 44.88% of males and 55.12% of females. The mean age of all students was 22.43 ± 1.68 years. Most students were single (85.72%). Overall, 24.87% had previously failed during their studies. However, the last known GPA was mostly A (43.49%) and only 35 students (0.59%) had a last known GPA of F. Most students belonged to a medical college that implemented a Problem-Based learning scheme (PBL) (65.84%), and only 16.50% of the total respondents were in private medical colleges. According to the Social Phobia Inventory severity score, 49.05% were not found to have SAD, while 20.22% were considered as mild, 18.32% as moderate, 8.21% severe and 4.21% as very severe (Table 1).

**Table 1:** Overall characteristics of Saudi Medical students (*N* = 5896)

Characteristics	N (%) 5896 (100)
**Age (µ, SD)**	22.43 (1.68)
**Sex**	
Males	2646 (44.88)
Females	3250 (55.12)
**Marital status**	
Single	5054 (85.72)
Married	637 (10.80)
Divorced	148 (02.51)
Widowed	57 (0.97)
**Total family income**	
< 10,000 SR	2091 (35.46)
10,000 – 20,000	2238 (37.96)
> 20,000	1567 (26.58)
**Year of study**	
Second year	1089 (18.47)
Third year	1027 (17.42)
Fourth year	1200 (20.35)
Fifth year	1063 (18.03)
Sixth year	819 (13.89)
Internship	698 (11.84)
**Previously failed**	
No	4431 (75.15)
Yes	1465 (24.85)
**Last known GPA**	
A	2564 (43.49)
B	1886 (31.99)
C	1158 (19.64)
D	253 (04.29)
F	35 (0.59)
**Method of college teaching**	
Problem based learning	3882 (65.84)
Non-Problem based or Traditional learning	2014 (34.16)
**Type of college**	
Governmental	4923 (83.50)
Private	973 (16.50)
**Social Phobia Inventory score**	
None	2892 (49.05)
Mild	1192 (20.22)
Moderate	1080 (18.32)
Severe	484 (08.21)
Very severe	248 (04.21)

**Fig. 1:**
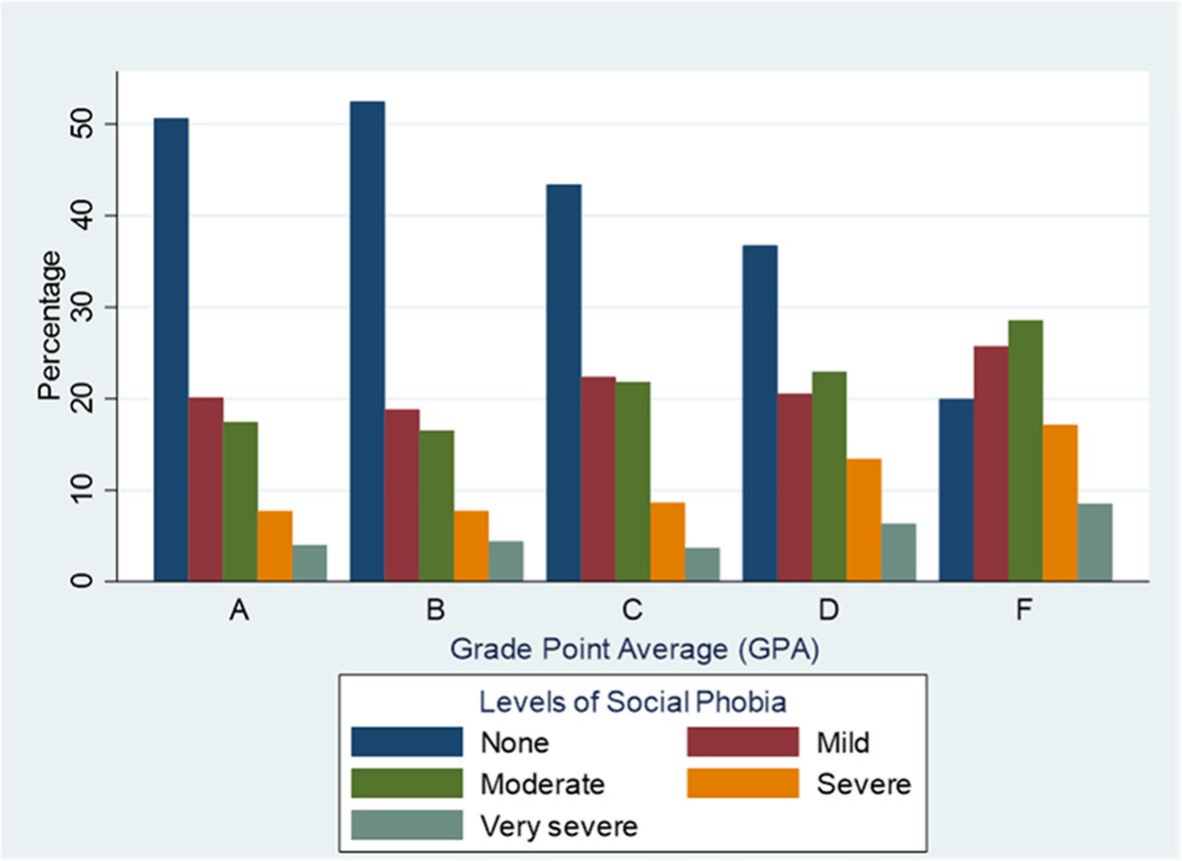
The five-level score of the Social Phobia Inventory and students’ GPA, Saudi Arabia, *N* = 5896

Figure 1 presents the five-level severity score of the Social Phobia Inventory across the different GPAs of the students. Among those with a GPA of A and B, a larger portion of the students are seen to not have (SAD). Whereas among those with a GPA of F, students were found to have a higher portion of (SAD) across all levels of severity, mild, moderate, severe, and very severe.

### Factors relating to the presence/absence of social anxiety disorder (SAD)

The presence of SAD was found to be associated with several factors at the bivariate analyses level (Table 2). For example, it was found to be associated with age (*P* < 0.01). It was also found to be statistically associated with sex (*P* = 0.02) where females were found to have more SAD compared to males. Previous academic failure and the last known GPA were highly statistically associated with SAD (*P* < 0.001). The data clearly shows that the lower the GPA the more the proportion of SAD (P for trend < 0.001). Neither family income nor the year of study were statistically associated with SAD in the study sample.

**Table 2:** Characteristics of medical students according to the Social Phobia Inventory score, Saudi Arabia, *N* = 5896

Characteristics	Social Phobia Inventory		*P*-value
	**Absent** **N (%)** **2892 (49.05)**	**Present** **N (%)** **3004 (50.94)**	
**Age (µ, SD)**	22.53 (1.63)	22.34 (1.72)	< 0.001
**Sex**			0.03
Males	1338 (50.57)	1308 (49.43)	
Females	1554 (47.82)	1696 (52.18)	
**Marital status**			0.02
Single	2487 (49.21)	2567 (50.79)	
Married	325 (51.02)	312 (48.98)	
Divorced	57 (38.51)	91 (61.49)	
Widowed	23 (40.35)	34 (59.65)	
**Total family income**			0.09
< 10,000 SR	1019 (48.73)	1072 (51.27)	
10,000 – 20,000	1069 (47.77)	1169 (52.23)	
> 20,000	804 (51.31)	763 (48.69)	
**Year of study**			0.11
Second year	499 (45.82)	590 (54.18)	
Third year	491 (47.82)	536 (52.19)	
Fourth year	606 (50.50)	594 (49.50)	
Fifth year	537 (50.52)	526 (49.84)	
Sixth year	420 (51.28)	399 (48.72)	
Internship	339 (48.57)	359 (51.43)	
**Previously failed**			< 0.001
No	2308 (52.09)	2123 (47.91)	
Yes	584 (39.86)	881 (60.14)	
**Last known GPA**^**‡**^			< 0.001
A	1299 (50.66)	1265 (49.34)	
B	990 (52.49)	896 (47.51)	
C	503 (43.44)	655 (56.56)	
D	93 (36.76)	160 (63.24)	
F	07 (20.00)	28 (80.00)	
**Method of college teaching**			< 0.001
Problem based learning	1979 (50.98)	1903 (49.02)	
Non-Problem based or Traditional learning	913 (45.33)	1101 (54.67)	
**Type of college**			< 0.001
Governmental	2486 (50.50)	2437 (49.50)	
Private	406 (41.73)	567 (58.27)	

^*‡*^* P for trend value* < *0.001*

### Factors associated with SAD according to multivariable analyses

Table 3 shows the results of the binary logistic regression both before and after adjustment. Age was a significant predictor whereby the risk of SAD decreased with increasing age both before and after adjustment (Unadjusted OR = 0.93, 95% CI = 0.90 – 0.96 and Adjusted OR = 0.92, 95% CI = 0.89 – 0.96 respectively). The model also showed that females were significantly more likely to have SAD when compared to males after adjustment (Adjusted OR = 1.46, 95% CI = 1.26 – 1.69). Having previously failed was also associated both before and after adjustment (Unadjusted OR = 1.64, 95% CI = 1.45 – 1.84 and Adjusted OR = 1.46, 95% CI = 1.26 – 1.69). An increase in risk was found with decreased GPA levels, for example the highest odds of 4.13 was found for students with a GPA of F (95% CI = 1.56 – 10.92) when compared to students with a GPA of A. Elevated risk was also observed for students who are enrolled in colleges that do not adopt a problem-based educational scheme and those who are in private colleges (Adjusted OR = 1.29, 95% CI = 1.15 – 1.46 and Adjusted OR = 1.29, 95% CI = 1.09 – 1.52).

**Table 3:** Unadjusted and adjusted Odds ratios of SAD among medical students, Kingdom of Saudi Arabia, *N* = 5896

Characteristics	Unadjusted OR	95% CI	Adjusted OR	95% CI
**Age (µ, SD)**	0.93*	0.90 – 0.96	0.92*	0.89 – 0.96
**Sex**				
Males	Ref			
Females	1.11	1.00 – 1.23	1.13*	1.01—1.26
**Previously failed**				
No	Ref			
Yes	1.64*	1.45 – 1.84	1.46*	1.26- 1.69
**Last known GPA**				
A	Ref			
B	0.92	0.82 – 1.04	0.94	0.82 – 1.08
C	1.33*	1.16 – 1.53	1.21*	1.03 – 1.43
D	1.76*	1.35 – 2.30	1.62*	1.19 – 2.21
F	4.10*	1.78 – 9.43	4.13*	1.56 – 10.92
**Method of college teaching**				
Problem based learning	Ref			
Non-Problem based or Traditional learning	1.24*	1.11 – 1.37	1.29*	1.15 – 1.46
**Type of college**				
Governmental	Ref			
Private	1.42*	1.23 – 1.63	1.29*	1.09—1.52

^*^P < 0.05

The model was highly significant (*P* < 0.001) with a Pseudo R^2^ value of 0.16. The Hosmer–Lemeshow value for this model was 11.25, with a *p*-value of 0.19 indicating good model fit.

## Discussion

The present study demonstrated that about half of the examined medical students in Saudi Arabia screened positive for SAD. Moreover, 8.21% and 4.21% of students had severe and very severe SAD symptoms, respectively. Other studies worldwide have also investigated the prevalence of SAD in undergraduate universities and medical students. Nevertheless, comparing our findings with these studies is difficult because of variations in the methodologies, study tools used, participants' backgrounds, social factors, and cultures. In agreement with the findings from the current study, Al-Hazmi et al., conducted a study among 504 medical students from Taibah university, Saudi Arabia, using the SPIN questionnaire and reported that 13.5% of the participating medical students had severe to very severe SAD [21]. Findings from the present study were higher than Desalegn et al.'s study which demonstrated that 31.2% (95% CI 27.3 to 35.6%) of undergraduate health science students in Ethiopia had SAD symptoms [24]. A study among 525 medical students in Germany revealed that 12.2% reported SAD symptoms [25]. In Iran, Afshari surveyed 400 medical sciences students using the SPIN tool and demonstrated that 41.5% and 13.2% of students had moderate and high SAD, respectively [26].

Furthermore, the findings of this study showed that SAD is less common among older aged students, which is consistent with Al-Hazmi et al. findings [21]. The decreased prevalence in older students may be attributed to their exposure to the clinical settings, as senior students tend to interact more with patients and are more experienced in interviewing skills. For instance, Alotaibi et al., found that older aged groups and higher-level students showed a higher score on the positive attitude scale towards learning communication skills [27]. Moreover, Davis et al.'s study showed that the final-year students had better communication skills than first-year students, indicating that they have a better vision and understanding of the importance of communication skills [28].

An expected and true finding of the current study is that social anxiety rates are higher among females compared to males. This finding is relevant to the (DSM-5) statement, which revealed that the prevalence of SAD is higher in females, and this difference is more pronounced among adolescents [4]. A similar finding was obtained by Xu et al.'s data survey from the National Epidemiologic Sample on Alcohol and Related Conditions among the United States adult population where the lifetime prevalence of SAD was higher in females than in males (5.7% and 4.2% respectively) [29]. Additionally, studies among the Canadian and European populations have shown similar results [30–34]. This outcome is contrary to Elhadad et al.'s study on only 380 medical students in Abha, Saudi Arabia, which found that SAD rates were higher among males. However, the Elhadad study population was obtained from a single institution and a relatively small sample size, hence, their results are less generalizable [35]. A possible explanation of why females are at higher risk of developing SAD can be best understood from a “vulnerability-stress perspective”. Exposure to variable psychosocial stressors and an increased biological and psychological vulnerability towards anxiety in females may explain the sex differences in anxiety disorders [36]. Interestingly, the current study found higher SAD rates among divorced, widowed, and singles than married ones. This finding supports the result of the systematic review conducted by Toe et al., which found that SAD was consistently associated with social isolation, such as being unmarried or living alone. Whether social isolation causes social anxiety or vice versa is still unclear [37].

Moreover, this study demonstrates that students who were enrolled in institutions implementing traditional teaching methods had an increased risk of having phobia compared to the students in PBL institutions, which indicates the effect of different learning styles on students’ mental well-being [38]. Furthermore, it draws attention to the nature of PBL, which revolves around the idea that a problem is of crucial importance in learning. It focuses on community problems, scientific problems, and real-life scenarios, motivating trainees and boosting their confidence. PBL promotes a deep learning approach rather than a superficial one by making trainees interact with information in a multilevel fashion. The absence of a teacher role in PBL increases the sense of responsibility towards self-learning and promotes personal development [39]. In other words, PBL is student-centered and encourages communication and teamwork through multiple tools of assessments, including presentations, small group discussions, seminars, assignments, and Objective Structured Clinical Examinations. The repeated exposure to social interactions and public speaking through PBL may increase students' confidence in social and clinical settings [40].

Many studies have reported high levels of stress and psychological comorbidities among Saudi medical students [41]. However, studies examining the differences between governmental and private medical schools in Saudi Arabia are limited. Moreover, we propose that the differences in teaching and learning approaches could explain finding a lower risk of SAD among governmental college students than those in private colleges. AlOmar et al. conducted a survey among 3767 students using the Approaches and Study Skills Inventory for Students (ASSIST), which showed that the deep and strategic approaches were predominant among Saudi medical students. In addition, private medical school students were more likely to adopt a strategic rather than a deep learning approach [42], which suggests that the difference in SAD levels between governmental and private medical college students may be explained by the differences in learning methods.

SAD was found to be associated with impairment in education and work productivity [43, 44]. A large cohort, population-based study in Sweden showed an inverse association between SAD and academic performance at different levels [43]. In line with this finding, the current study revealed that a lower GPA was linked to a higher risk of SAD; hence it was more frequently reported among students with a previous failure in medical school. Furthermore, previous Saudi studies have also reported a similar inverse relationship [21, 35]. This may be explained by the fact that medical school environments are highly competitive; students are working hard to achieve higher grades and GPA to look for opportunities in the postgraduate residency programs and jobs. These stressors make medical students vulnerable to mental health problems [45]. Moreover, the presence of students with low GPA or previous failure with their high achieving colleagues could be another burden on them. It might lead to social isolation, low self-esteem, being inactive in the group work, and consequently having social anxiety symptoms more frequently than their peers.

To the best of our knowledge, the current study is the first Saudi study investigating SAD among a large sample of medical students from all regions in the kingdom. However, some limitations exist. Firstly, the sample only included medical students and did not represent the general Saudi population. Secondly, since the design of the study was cross-sectional, temporality and causality between factors could not be assured. Additionally, despite of the high response rate the possibility of response bias could not be eliminated. Finally, the SPIN tool utilized in the study is a screening tool, and the high-risk cases need a further diagnostic step by a clinical interview.

## Conclusion

The current study found that SAD was highly prevalent among the investigated medical students in Saudi Arabia. Older students had lower odds of SAD. On the other hand, being female, studying in private colleges or with non-problem-based learning methods, and having a history of a previous failure in the medical school or a lower GPA were identified as factors that had higher odds of SAD. These findings emphasize the positive role of the university faculty members, counselors, and mentors in supporting these students and encouraging them to participate in curricular and extracurricular activities. In addition, evaluation of the educational environment and the types of the teaching curriculum in Saudi Medical schools is necessary to optimize students learning experience and maintain their psychological wellbeing. Along with enhancing the primary care providers and mental health care experts to accomplish their role of early detection and management of these cases.

## Data Availability

The datasets generated and analysed for the current study are not publicly available for data protection reasons. However, the data that support the findings of this study may be available from the corresponding author on reasonable request.

## Abbreviations

SAD: Social Anxiety Disorder
DSM-5: Diagnostic and Statistical Manual of Mental Disorders
KSA: Kingdom of Saudi Arabia
GPA: Grade Point Average
PBL: Problem-Based Learning
ASSIST: Approaches and Study Skills Inventory for Students
